# Meta‐Analysis of the Diagnostic Efficiency of THSD7A‐AB for the Diagnosis of Idiopathic Membranous Nephropathy

**DOI:** 10.1002/gch2.201900099

**Published:** 2020-09-06

**Authors:** Yipeng Liu, Shanshan Zheng, Chaoqun Ma, Ying Lian, Xiaoli Zheng, Peizhong Guan, Baobao Wang, Xiaojie Gong, Feng Gao, Liming Liang, Dongmei Xu

**Affiliations:** ^1^ Department of Nephrology The First Affiliated Hospital of Shandong First Medical University & Shandong Provincial Qianfoshan Hospital Jinan China; ^2^ Department of Emergency Shandong Provincial Hospital Affiliated to Shandong First Medical University Jinan China; ^3^ Department of Medical Record Management The First Affiliated Hospital of Shandong First Medical University & Shandong Provincial Qianfoshan Hospital Jinan China; ^4^ Department of Nephrology YEDA Hospital Yantai China; ^5^ Shandong Provincial Key Laboratory for Rheumatic Disease and Translational Medicine Jinan China; ^6^ Nephrology Research Institute of Shandong Province Jinan China

**Keywords:** diagnostic efficiency, idiopathic membranous nephropathy, meta‐analysis

## Abstract

Thrombospondin type I domain‐containing 7A (THSD7A), is a specific autoantigen of adult idiopathic membranous nephropathy (IMN), whose circulating antibody (THSD7A‐AB) represents a promising biomarker for diagnosis of IMN. The objective of this meta‐analysis is to investigate the diagnostic efficiency of THSD7A‐AB for IMN. After rigorous data extraction, quality assessment, and data analysis, 10 articles (4545 patients) are included. For IMN, the summary sensitivity is 4% (2–7%), and the specificity is 99% (98–100%). The summary positive likelihood ratio (PLR) and negative likelihood ratio (NLR) are 5.40 (2.40–11.90) and 0.97 (0.95–0.99), respectively. The diagnostic odds ratio (DOR) is 6.00 (2.00–12.00). The area under the summary receiver operating characteristic curve (AUC) is 0.78 (0.74–0.81). For M‐type phospholipase A2 receptor (PLA2R)‐negative IMN, the summary sensitivity is 8% (6–10%), specificity is 100% (99–100%). The summary PLR and NLR are 15.80 (5.70–44.00) and 0.93 (0.91–0.95), respectively. The DOR is 17.00 (6.00–48.00). The AUC is 0.99 (0.98–1.00). THSD7A‐AB has higher diagnostic value in PLA2R‐negative patients than in IMN patients. These results suggest that THSD7A‐AB could possibly be applied as an auxiliary non‐invasive diagnostic method for PLA2R‐negative IMN.

## Graphical abstract

The present meta‐analysis systematically assesses the diagnostic efficiency of THSD7A‐AB testing in patients with adult idiopathic membranous nephropathy (IMN) for the first time. It is found that thrombospondin type I domain‐containing 7A (THSD7A)‐AB has a higher diagnostic value for M‐type phospholipase A2 receptor (PLA2R)‐negative patients than for IMN patients. THSD7A‐AB testing can be applied as an auxiliary non‐invasive diagnostic method for PLA2R‐negative IMN.

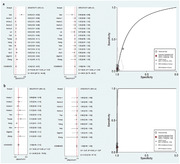

## 1 Introduction

Membranous nephropathy, which has idiopathic and secondary forms, is one of the leading causes of adult nephrotic syndrome.^[^ ^1^ ^]^ Except for a few secondary cases due to viral infection, autoimmune diseases, thyroiditis, malignancy, and/or drug abuse, ≈80% of all cases of membranous nephropathy are referred to as idiopathic membranous nephropathy (IMN) because they have unknown etiology.^[^ ^2^ ^]^ There are significant differences in the treatment of the two forms of diseases; the Kidney Disease: Improving Global Outcomes guidelines recommend corticosteroids combined with calcineurin inhibitors/alkylating agents as the initial therapy for IMN.^[^ ^3^ ^]^ However, the treatment of secondary membranous nephropathy (SMN) is mainly focused on the etiology. Given the limitations of traditional renal biopsy diagnosis, such as perirenal hematoma, arteriovenous fistulas, infection, and damage to other organs,^[^ ^4^ ^]^ it is extremely important to find reliable serological biomarkers to differentiate between IMN and SMN.

In 2009, M‐type phospholipase A2 receptor (PLA2R) was identified as the first target antigen for IMN,^[^ ^5^ ^]^ and the circulating antibody against PLA2R (PLA2R‐AB) was used for the non‐invasive diagnosis of IMN, with 78% sensitivity and 99% specificity.^[^ ^6^ ^]^ Thrombospondin type I domain‐containing 7A (THSD7A), which is similar to PLA2R in structure, was identified as a second autoantigen of adult IMN.^[^ ^7^ ^]^ Several studies have indicated that the circulating THSD7A‐AB levels represent another promising alternative biomarker for the diagnosis of IMN.

Serological testing for circulating THSD7A‐AB provides a rapid IMN diagnostic method for clinicians. However, the reported diagnostic efficiency of THSD7A‐AB has been extremely varied among different studies. For example, the sensitivity of THSD7A‐AB tests ranged from 0% to 35%, and the specificity ranged from 90% to 100%.^[^ ^7^, ^8^, ^9^, ^10^, ^11^, ^12^, ^13^, ^14^, ^15^, ^16^ ^]^ Although THSD7A‐AB may be a new biomarker for IMN diagnosis, its efficiency is still controversial. Therefore, we performed the present meta‐analysis to comprehensively assess the diagnostic efficiency of THSD7A‐AB testing in patients with IMN.

For the sake of clarity, **Table** **1** presents the full list of abbreviations used in this meta‐analysis.

**Table 1:** List of abbreviations used in this meta‐analysis

Abbreviation	Full name
THSD7A	Thrombospondin type I domain‐containing 7A
IMN	Idiopathic membranous nephropathy
PLR	Positive likelihood ratio
NLR	Negative likelihood ratio
DOR	Diagnostic odds ratio
AUC	Area under the summary receiver operating characteristic curve
PLA2R	M‐type phospholipase A2 receptor
SMN	Secondary membranous nephropathy
PLA2R‐AB	Antibody against M‐type phospholipase A2 receptor
THSD7A‐AB	Antibody against thrombospondin type I domain‐containing 7A
Wanfang	Digital Journal of Wanfang Data
VIP	VIP Database for Chinese Technical Periodicals
CNKI	Chinese National Knowledge Infrastructure
QUADAS‐2	Quality Assessment of Diagnostic Accuracy Studies‐2
SROC	Summary receiver operating characteristic
IFT	Immunofluorescence test

## 2 Results

### 2.1 Study Characteristics

**Figure 1:**
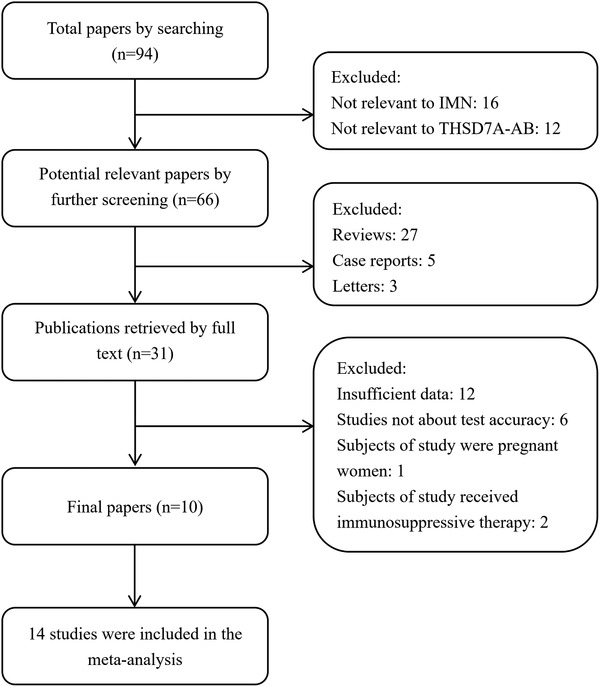
Flowchart of literature search.

As shown in **Figure** **1**, there were 94 papers found in our primary search. Twenty‐eight were excluded (16 articles were not relevant to IMN, 12 articles were on the presence of THSD7A in renal tissue). Therefore, there were 66 potentially relevant articles found in our search. Thirty‐five articles met the exclusion criteria (27 reviews, 3 letters, and 5 case reports). The remaining 31 articles were retrieved for full‐text review. Twenty‐one articles were excluded (6 articles did not investigate the test accuracy, 12 articles did not provide sufficient data, 1 article only investigated THSD7A‐AB in pregnant woman and the subjects of 2 articles received immunosuppressive therapy). Finally, 10 articles, including 14 studies, were included in the present meta‐analysis. The characteristics of the selected studies are shown in **Table** **2**. The total population of the studies was 4545. THSD7A‐AB was detectable by Western blotting in six studies, by ELISA method in five studies and by IFT method in three studies. Six studies had an interval between the index test and the renal biopsy, and eight studies did the index test and the renal biopsy simultaneously. Six studies used patients with SMN as controls, 12 studies used patients with other glomerular diseases as controls, and 3 studies used healthy subjects as controls.

**Table 2:** Characteristics of the included eligible studies

Year	Study	Region	Method	Funding	Test interval	Case	Control1	Control2	Control3	TP	FP1	FP2	FP3	FN	TN1	TN2	TN3
2017	Gao^[^ ^13^ ^]^	Asia	ELISA	No	No	40	10	10		14	2	0		26	8	10	
2017	Hoxha 1^[^ ^11^ ^]^	Europe	IFT	Government	Yes	345		47		8		0		337		47	
2017	Hoxha 2^[^ ^11^ ^]^	Europe	WB	Government	Yes	345		47		8		0		337		47	
2017	Hoxha 3^[^ ^11^ ^]^	Europe	WB	Government	Yes	192		47		9		0		183		47	
2017	Hoxha 4^[^ ^11^ ^]^	America	WB	Government	Yes	125		47		5		0		120		47	
2019	Tian^[^ ^9^ ^]^	Asia	IFT	Government	No	212	118	84		6	2	0		206	116	84	
2016	Lin^[^ ^10^ ^]^	Asia	WB	Government	No	99	37			2	1			97	36		
2014	Tomas^[^ ^7^ ^]^	Europe	WB	Government	Yes	118	35	76	44	6	1	0	0	112	34	76	44
2017	Wang^[^ ^12^ ^]^	Asia	IFT	Government	No	578	114	64	20	8	1	0	0	570	113	64	20
2016	Wen^[^ ^8^ ^]^	Asia	ELISA	No	No	86	30	20		0	0	0		86	30	20	
2017	Xia 1^[^ ^14^ ^]^	Asia	WB	Government	No	127		50		9		0		118		50	
2017	Xia 2^[^ ^14^ ^]^	Asia	ELISA	Government	No	127		50		10		1		117		49	
2019	Zaghrini^[^ ^15^ ^]^	Europe	ELISA	Government	Yes	1012			52	28			0	984			52
2018	Zhang^[^ ^16^ ^]^	Asia	ELISA	No	No	114		23		11		0		103		23	

1: SMN control, 2: other glomerular disease control, 3: healthy control. Test interval: yes indicates testing after the biopsy; no indicates testing simultaneous with the biopsy. Hoxha 1 indicates the Prospective Hamburg cohort with IFT method in Hoxha's study. Hoxha 2 indicates the Prospective Hamburg cohort with WB method in Hoxha's study. Hoxha 3 indicates the Retrospective Hamburg cohort with WB method in Hoxha's study. Hoxha 4 indicates the Retrospective Boston cohort with IFT method in Hoxha's study. Xia 1 indicates the WB testing results in Xia's study. Xia 2 indicates the ELISA testing results in Xia's study.

### 2.2 Methodological Quality of the Included Studies

**Figure 2:**
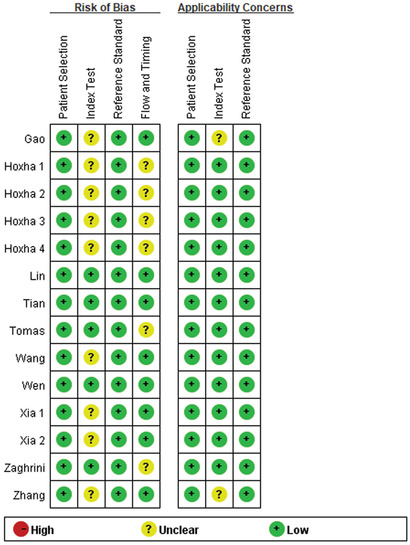
The QUADAS‐2 results for selected studies.

According to the QUADAS‐2, the quality assessment of the selected studies is shown in **Figure** **2**. Although the overall quality of the eligible studies was robust, six studies had unclear risk regarding the flow and timing, which was derived from the time interval between index test and reference standard. Meanwhile, nine studies showed unclear risk regarding the index test that was caused by the awareness of the results of the reference standard.

### 2.3 Meta‐Analysis

#### 2.3.1 Diagnostic Efficiency in IMN

**Figure 3:**
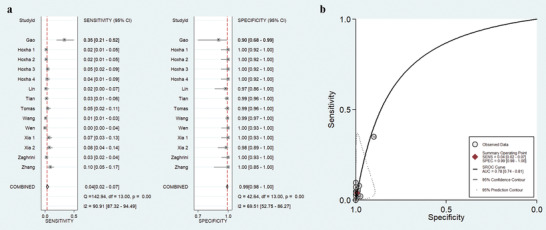
Forest plots (a) and summary receiver operating characteristic curve (b) of the diagnostic efficiency of THSD7A‐AB for IMN patients.

In our meta‐analysis, Spearman's correlation coefficient was 0.006 (*p* = 0.971), which suggested that there was no threshold effect among these eligible studies; then, a random effect model was selected to analyze the accuracy of THSD7A‐AB for IMN diagnosis. As shown in **Figure** **3**, the pooled sensitivity was 4% (95% CI: 2–7%), and the pooled specificity was 99% (95% CI: 98–100%). Meanwhile, *I^2^* was 90.91% (*P* < 0.01) for the pooled sensitivity, suggesting high heterogeneity in the sample of studies. The pooled positive likelihood ratio (PLR) was 5.40 (95% CI: 2.40–11.90), the pooled negative likelihood ratio (NLR) was 0.97 (95% CI: 0.95–0.99), and the pooled diagnostic odds ratio (DOR) was 6.00 (95% CI: 2.00–12.00). The summary receiver operating characteristic (SROC) graph with the 95% confidence contour and the 95% prediction contour is shown in Figure 3b, the area under the summary receiver operating characteristic curve (AUC) was 0.78 (95% CI: 0.74–0.81), indicating a relatively acceptable level of summary diagnostic accuracy of THSD7A‐AB in IMN patients.

#### 2.3.2 Subgroup and Sensitivity Analysis

A subgroup analysis was then carried out to determine the cause of heterogeneity. As shown in **Table** **3**, the diagnostic accuracy of THSD7A‐AB detection was higher when the Western blotting method was used than when the ELISA method was used. Similar findings were also found in the following factors: large sample size, studies with an interval between the index test and the renal biopsy, and studies using patients with other glomerular disease as controls.

**Table 3:** Subgroup analysis for the accuracy of THSD7A‐AB for IMN detection

Subgroup	*N*	Sensitivity	Specificity	Positive likelihood ratio	Negative likelihood ratio	AUC
Method						
Western blotting	6	0.04 (0.03–0.06)	0.99 (0.98–1.00)	7.60 (1.70–34.20)	0.97 (0.95–0.98)	0.72 (0.68–0.76)
IFT	3	–	–	–	–	–
ELISA	5	0.06 (0.02–0.19)	0.99 (0.91–1.00)	8.30 (1.00–71.10)	0.95 (0.89–1.02)	0.68 (0.64–0.72)
Region						
Europe	5	0.03 (0.02–0.04)	1.00 (0.98–1.00)	10.20 (1.40–73.40)	0.97 (0.96–0.98)	0.83 (0.80–0.86)
America	1	–	–	–	–	–
Asia	8	0.04 (0.02–0.11)	0.99 (0.97–1.00)	3.60 (1.60–8.50)	0.97 (0.93–1.00)	0.83 (0.79–0.86)
Sample size						
>200	7	0.03 (0.02–0.03)	0.99 (0.98–1.00)	4.90 (1.80–13.40)	0.98 (0.97–0.99)	0.99 (0.97–0.99)
≤200	7	0.06 (0.02–0.14)	0.99 (0.95–1.00)	5.50 (1.50–21.20)	0.95 (0.91–1.00)	0.76 (0.72–0.79)
Interval						
No	8	0.04 (0.02–0.11)	0.99 (0.97–1.00)	3.60 (1.60–8.50)	0.97 (0.93–1.00)	0.83 (0.79–0.86)
After biopsy test	6	0.03 (0.02–0.04)	1.00 (0.98–1.00)	11.80 (1.60–85.00)	0.97 (0.96–0.98)	0.85 (0.81–0.87)
Control						
SMN	6	0.03 (0.01–0.10)	0.98 (0.93–0.99)	1.50 (0.60–3.80)	0.99 (0.96–1.02)	0.65 (0.61–0.69)
Other glomerular disease	12	0.04 (0.02–0.08)	1.00 (0.98–1.00)	30.4 (2.70–339.20)	0.96 (0.94–0.98)	0.86 (0.83–0.89)
Health	3	–	–	–	–	–

Subsequently, we performed a sensitivity analysis to evaluate the effect of individual studies on the pooled diagnostic accuracy of THSD7A‐AB testing for IMN. As shown in Table S1 (Supporting Information), the diagnostic accuracy of THSD7A‐AB testing for IMN was relatively stable after the removal of each individual study.

#### 2.3.3 Publication Bias Assessment

The publication bias of the selected studies was assessed by the Deeks’ funnel plot asymmetry test. The funnel plot with a superimposed regression line is shown in Figure S1 (Supporting Information). The result suggested no publication bias among studies (*P *= 0.92).

#### 2.3.4 Diagnostic Efficiency in PLA2R‐Negative IMN

**Figure 4:**
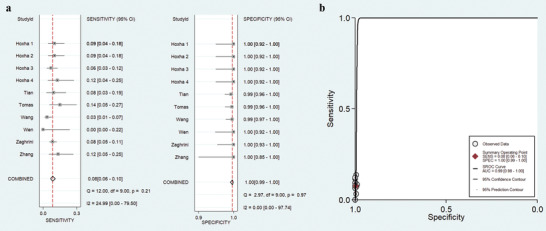
Forest plots (a) and summary receiver operating characteristic curve (b) of the diagnostic efficiency of THSD7A‐AB for PLA2R‐negative IMN patients.

A further analysis was performed to evaluate the diagnosis accuracy of THSD7A‐AB for the diagnosis of PLA2R‐negative IMN cases. The characteristics of the included studies are shown in **Table** **4**. And as shown in **Figure** **4**, the pooled sensitivity and specificity of THSD7A‐AB was 8% (95% CI: 6–10%) and 100% (95% CI: 99–100%), respectively. The summary PLR was 15.80 (95% CI: 5.70–44.00), the summary NLR was 0.93 (95% CI: 0.91–0.95), and the summary DOR was 17.00 (95% CI: 6.00–48.00). The SROC graph with the 95% confidence contour and the 95% prediction contour is shown in Figure 4b, the AUC was 0.99 (95% CI: 0.98–1.00), indicating that the summary diagnostic accuracy of THSD7A‐AB in PLA2R‐negative patients is better than in IMN patients.

**Table 4:** Characteristics of the included eligible studies about PLA2R‐AB‐negative IMN cases

Year	Study	Region	Method	Funding	Test interval	Case	Control1	Control2	Control3	TP	FP1	FP2	FP3	FN	TN1	TN2	TN3
2017	Hoxha 1^[^ ^11^ ^]^	Europe	IFT	Government	Yes	86		47		8		0		78		47	
2017	Hoxha 2^[^ ^11^ ^]^	Europe	WB	Government	Yes	86		47		8		0		78		47	
2017	Hoxha 3^[^ ^11^ ^]^	America	WB	Government	Yes	141		47		9		0		132		47	
2017	Hoxha 4^[^ ^11^ ^]^	Europe	WB	Government	Yes	43		47		5		0		38		47	
2019	Tian^[^ ^9^ ^]^	Asia	IFT	Government	No	59	107	84		5	2	0		54	105	84	
2014	Tomas^[^ ^7^ ^]^	Europe	WB	Government	Yes	44	35	76	44	6	1	0	0	38	34	76	44
2017	Wang^[^ ^12^ ^]^	Asia	IFT	Government	No	184	85	64	20	6	1	0	0	178	84	64	20
2016	Wen^[^ ^8^ ^]^	Asia	ELISA	No	No	15	26	20		0	0	0		15	26	20	
2019	Zaghrini^[^ ^15^ ^]^	Europe	ELISA	Government	Yes	325			52	25			0	300			52
2018	Zhang^[^ ^16^ ^]^	Asia	ELISA	No	No	49		23		6		0		43		23	

1: SMN control, 2: other glomerular disease control, 3: healthy control. Test interval: yes indicates testing after the biopsy; no indicates testing simultaneous with the biopsy. Hoxha 1 indicates the Prospective Hamburg cohort with IFT method in Hoxha's study. Hoxha 2 indicates the Prospective Hamburg cohort with WB method in Hoxha's study. Hoxha 3 indicates the Retrospective Hamburg cohort with WB method in Hoxha's study. Hoxha 4 indicates the Retrospective Boston cohort with IFT method in Hoxha's study.

## 3 Discussion

Since THSD7A was discovered as the second antigenic target of autoimmune IMN, a more in‐depth understanding of the pathogenesis of this disease has been achieved. In addition to the classic complement activation pathway that leads to sublethal podocyte injury, recent studies^[^ ^17^, ^18^ ^]^ have indicated that the THSD7A antigen‐antibody immune complex in situ might directly interfere with podocyte integrity, leading to glomerular filtration barrier disruption and proteinuria. THSD7A‐AB detection shows fairly good application prospects in multiple aspects of IMN, such as diagnosis, disease activity, clinical outcome, and treatment efficacy.^[^ ^12^, ^19^ ^]^ Despite this, the diagnostic efficiency of THSD7A‐AB for IMN is still controversial. Ren et al.'s systematic review published in 2018 firstly described the prevalence of THSD7A in IMN patients, however, the diagnostic efficiency of THSD7A in IMN was not systematically evaluated. Their results showed that the prevalence of THSD7A was 3% (95% CI: 3–4%) in IMN patients without the exclusion of subjects receiving immunosuppressive therapy and without the identification of antibody detection in the serum or antigen detection in the renal tissue.^[^ ^20^ ^]^ Given the actual clinical needs of non‐invasive diagnosis, our meta‐analysis firstly systematically reviewed the diagnostic efficiency of serum THSD7A‐AB testing in patients with IMN with no publication bias, and in view of the effect of immunosuppression on antibody titer, we excluded patients receiving immunosuppressive therapy and found that the summary sensitivity was 4% (95% CI: 2–7%) and the summary specificity was 99% (95% CI: 98–100%). Although it is not sensitive enough, it is very specific for the diagnosis of IMN.

The existence of heterogeneity among the studies may affect the diagnostic efficiency. The results of the subgroup analysis indicated that the heterogeneity may be derived from type of control, sample size, testing method, and time interval. Further analysis revealed that, in addition to the apparent effect of “control type” on the diagnostic efficiency, the test interval between THSD7A‐AB detection and renal biopsy was the significant source of heterogeneity. There are several possible explanations for why the testing interval was the significant source of heterogeneity found in our meta‐analysis. First, various stages of disease may have been achieved during the testing interval. Second, the effect of immunosuppressive therapy must also be taken into account. Third, the spontaneous remission of disease should also be taken into account. For example, the testing was performed between 0 and 87 months after renal biopsy in the study of Tomas et al.^[^ ^7^ ^]^ It is possible that the serum titers of THSD7A‐AB decreased in some patients who had entered into an immunologically inactive stage or even have achieved spontaneous remission. Therefore, we suggest that serological testing should be performed at the time of initial diagnosis, rather than a period of time after renal biopsy. This would avoid the possible confusion introduced by therapeutic intervention and prognosis assessment.

To further explore the clinical application value of serum THSD7A‐AB detection, we analyzed its value as a diagnostic biomarker in PLA2R‐negative IMN patients. Encouragingly, the evaluation indexes for diagnostic efficiency were significantly ameliorated. The summary sensitivity for PLA2R‐negative IMN increased by 100% compared to the sensitivity in all IMN patients without significantly changing the specificity. The summary PLR increased from 5.40 to 15.80, which suggested that its capacity for the correct identification of positive subjects was increased nearly 3 times. The summary NLR was reduced from 0.97 to 0.93, which suggested that its capacity for the correct identification of negative subjects was increased by 4%. The summary DOR was increased from 6.00 to 17.00, which suggested that its comprehensive diagnostic capacity was increased nearly 3 times. Collectively, the results suggested THSD7A‐AB has higher diagnostic value for PLA2R‐negative patients than for IMN patients, so we suggest that serum THSD7A‐AB detection should be combined with PLA2R‐AB to applied for the non‐invasive diagnosis of IMN. After the discovery of THSD7A, neural epidermal growth factor‐like 1 protein^[^ ^21^ ^]^ and Semaphorin 3B^[^ ^22^ ^]^ have also been recognized as the target antigens of IMN, their potential value as the diagnostic markers for IMN needs further systematic evaluation. We believe that with the discovery of more and more specific antigen‐antibody targets for IMN, the era of complete non‐invasive diagnosis of IMN will surely come.

A comprehensive literature search is the strength of our meta‐analysis, but there were still several limitations within this study that must be acknowledged. First, THSD7A‐AB is a newly discovered biomarker, therefore, more well‐designed studies are needed to verify our conclusions. Second, not all included articles were of high quality. For example, the majority of studies did not report whether the antibody examination was performed while the investigator was blind to the renal biopsy results. Such methodological limitations might have biased our final conclusions.

In conclusion, this is the first study that systematically assessed the diagnostic efficiency of THSD7A‐AB testing in patients with IMN. In addition, we found that THSD7A‐AB has a higher diagnostic value for PLA2R‐negative patients than for IMN patients. It could be applied as an auxiliary non‐invasive diagnostic method for PLA2R‐negative IMN. Considering our limitations and the heterogeneity among our chosen studies, well‐designed multicenter prospective studies with large sample sizes are needed to further validate the results of this meta‐analysis.

## 4 Experimental Section

##### Data Sources and Search Strategy

PubMed, Embase, ClinicalTrials, SinoMed, Digital Journal of Wanfang Data (Wanfang), VIP Database for Chinese Technical Periodicals (VIP), and Chinese National Knowledge Infrastructure (CNKI) were searched to identify eligible studies published prior to Nov. 1st, 2019. The search terms used were “thrombospondin type I domain‐containing 7A,” “THSD7A protein,” and “THSD7A.” Studies were also identified by manually searching the references cited in selected articles. No language restriction was imposed in the meta‐analysis. Two authors (Yipeng Liu and Xiaoli Zheng) independently determined the eligibility of the studies, and disagreements were resolved by discussion and consensus.

##### Study Selection

Studies included in the current meta‐analysis met the following criteria: (1) evaluated the accuracy of THSD7A‐AB testing for IMN diagnosis; (2) estimated the sensitivity and specificity of the THSD7A‐AB test; and (3) used renal biopsy results as the diagnostic gold standard. The exclusion criteria included: (1) case report, review, letter, editorial, or comment; (2) did not report test accuracy; (3) study subjects received immunosuppressive therapy; or (4) did not provide sufficient data. If studies had overlapping subjects, only the largest study was included.

##### Data Extraction and Quality Assessment

Two investigators (Yipeng Liu and Ying Lian) independently extracted the following data from all included articles: first author, year of publication, region, test method, funding source, the time interval between biopsy and THSD7A‐AB testing, sample size and evaluation indexes (true positive, false positive, true negative, and false negative). The extracted data were confirmed by the third investigator (Chaoqun Ma).

The methodological quality of studies was evaluated independently by two researchers (Shanshan Zheng and Ying Lian) with the 4‐component Quality Assessment of Diagnostic Accuracy Studies‐2 (QUADAS‐2).^[^ ^23^ ^]^ The four components were as follows: (1) patient selection, (2) index test, (3) reference standard, and (4) flow and timing. Each item of the component was assessed as “yes,” “no,” or “unclear.” When discrepancies in assessment existed, a consensus was reached.

##### Data Analysis

The Spearman's correlation coefficient was calculated to assess the threshold effect. A random effect model was used in the meta‐analysis to calculate the summary sensitivity, specificity, PLR, NLR, and DOR across studies. A SROC curve was then used to plot the consistency of results among all studies as well as the accuracy of the test, and the AUC was calculated. The heterogeneity among studies was evaluated with *I^2^* and chi‐square tests. The *I* ^2^ < 25% was considered as low heterogeneity, and *I* ^2^ > 75% as high heterogeneity. Subgroup analyses were carried out to identify the sources of heterogeneity. Such sources included the following: region (e.g., Europe, America, Asia), type of control (e.g., patients with SMN, patients with other glomerular diseases, healthy controls), sample size (>200 vs ≤200), testing method (e.g., Western blotting, immunofluorescence test (IFT), ELISA), and time interval (e.g., testing simultaneously with the biopsy versus testing after the biopsy). Sensitivity analysis was performed by omitting one study at a time to further evaluate the stability of the findings.

Deeks’ funnel plot asymmetry test was used to explore potential publication biases.^[^ ^24^ ^]^ All analyses were performed in mdias module in Stata 12.0 (College Station, TX, USA). A two‐sided *P* < 0.05 was defined as statistically significant, except for heterogeneity testing, which had a boundary level of 0.10.

## Conflict of Interest

The authors declare no conflict of interest.

## Supporting information

Supporting Information
