# Supplementary material for: Meta‐Analysis of the Diagnostic Efficiency of THSD7A‐AB for the Diagnosis of Idiopathic Membranous Nephropathy
Source: Glob Chall. 2020 Sep 6;4(11):1900099. doi: 10.1002/gch2.201900099 (PMC7607246; doi:10.1002/gch2.201900099)
Supplement: Supplementary file 1 — Supporting Information [file GCH2-4-1900099-s001.pdf]

© 2020 Wiley-VCH GmbH

Supporting Information

for *Global Challenges,* DOI: 10.1002/gch2.201900099

Meta-Analysis of the Diagnostic Efficiency of THSD7A-AB for the Diagnosis of Idiopathic Membranous Nephropathy

*Yipeng Liu, Shanshan Zheng, Chaoqun Ma, Ying Lian, Xiaoli Zheng, Peizhong Guan, Baobao Wang, Xiaojie Gong, Feng Gao, Liming Liang, and Dongmei Xu**

© 2020 Wiley-VCH GmbH

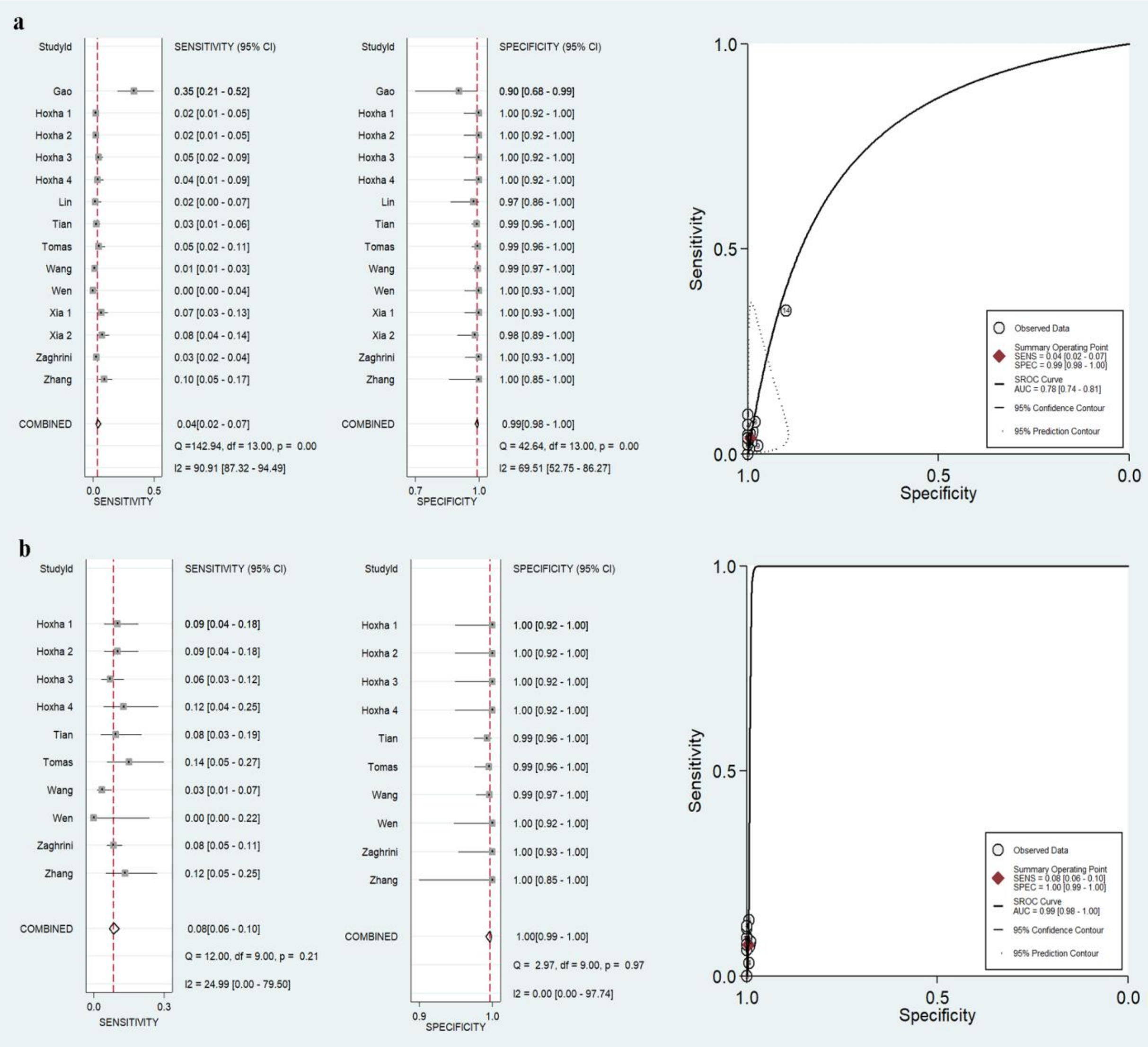

Supplementary Information

Figure S1 Deeks' Funnel Plot Asymmetry Test

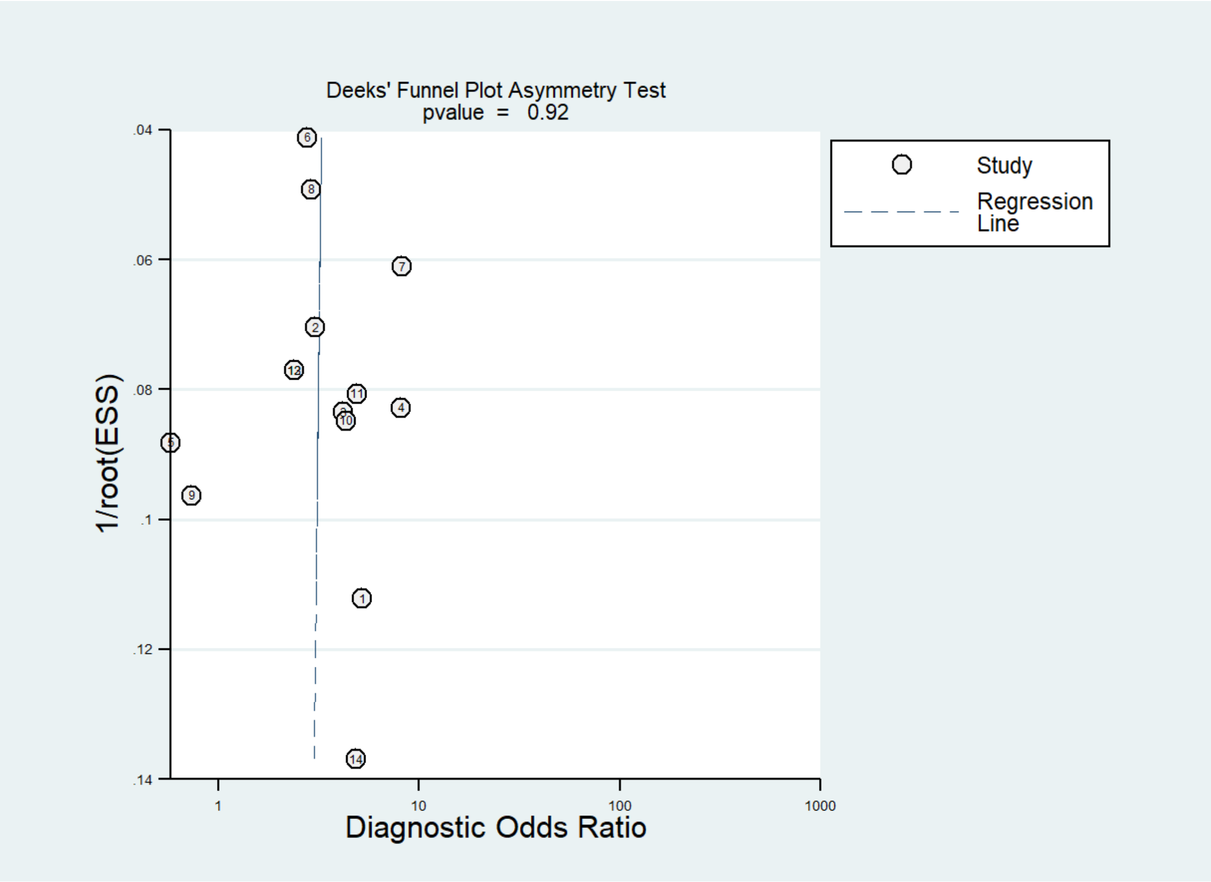
Deeks' Funnel Plot Asymmetry Test
pvalue = 0.92
1/root(ESS)
.04
.06
.08
.1
.12
.14
1
10
100
1000
Diagnostic Odds Ratio
Study
Regression Line

Table S1 Sensitivity analysis for the accuracy of THSD7A-AB test for IMN

Study omitted	Sensitivity	Specificity	Positive LikelihoodRatio	NegativeLikelihood Ratio	AUC
Gao	0.03 (0.02-0.05)	0.99 (0.99-1.00)	5.50 (2.40-13.00)	0.97 (0.96-0.98)	0.98 (0.97-0.99)
Hoxha 1	0.04 (0.02-0.07)	0.99 (0.98-1.00)	5.20 (2.40-11.40)	0.97 (0.95-0.99)	0.79 (0.75-0.82)
Hoxha 2	0.04 (0.02-0.07)	0.99 (0.98-1.00)	5.20 (2.40-11.40)	0.97 (0.95-0.99)	0.79 (0.75-0.82)
Hoxha 3	0.04 (0.02-0.07)	0.99 (0.98-1.00)	5.00 (2.30-11.20)	0.97 (0.95-0.99)	0.78 (0.75-0.82)
Hoxha 4	0.04 (0.02-0.07)	0.99 (0.98-1.00)	5.10 (2.30-11.20)	0.97 (0.95-0.99)	0.78 (0.75-0.82)
Tian	0.04 (0.02-0.07)	0.99 (0.98-1.00)	6.50 (2.50-17.30)	0.97 (0.95-0.99)	0.75 (0.71-0.79)
Lin	0.04 (0.02-0.07)	0.99 (0.98-1.00)	6.40 (2.60-15.30)	0.97 (0.95-0.98)	0.75 (0.71-0.79)
Tomas	0.04 (0.02-0.07)	0.99 (0.98-1.00)	5.10 (2.20-11.90)	0.97 (0.95-0.99)	0.77 (0.74-0.81)
Wang	0.04 (0.03-0.07)	0.99 (0.98-1.00)	6.10 (2.50-15.20)	0.96 (0.94-0.98)	0.73 (0.69-0.77)
Wen	0.04 (0.03-0.07)	0.99 (0.98-1.00)	5.30 (2.50-11.50)	0.96 (0.94-0.98)	0.78 (0.74-0.81)
Xia 1	0.04 (0.02-0.06)	0.99 (0.98-1.00)	4.90 (2.20-11.0)	0.97 (0.95-0.99)	0.77 (0.73-0.80)
Xia 2	0.04 (0.02-0.06)	0.99 (0.98-1.00)	5.60 (2.40-13.10)	0.97 (0.95-0.99)	0.79 (0.75-0.82)
Zaghrini	0.04 (0.02-0.07)	0.99 (0.98-1.00)	5.10 (2.30-11.30)	0.97 (0.95-0.99)	0.79 (0.75-0.83)
Zhang	0.04 (0.02-0.06)	0.99 (0.98-1.00)	5.10 (2.30-11.50)	0.97 (0.95-0.99)	0.76 (0.72-0.80)

Hoxha 1 indicatesthe Prospective Hamburg cohort with IFT method in Hoxha’s study. Hoxha 2 indicatesthe Prospective Hamburg cohort with WB method in Hoxha’s study. Hoxha 3 indicatesthe Retrospective Hamburg cohort with WB method in Hoxha’s study. Hoxha 4 indicatesthe Retrospective Boston cohort with IFT method in Hoxha’s study. Xia 1 indicatesthe WB testing results in Xia’s study. Xia 2 indicatesthe ELISA testing results in Xia’s study.

Checklist S1 PRISMA checklist. Preferred Reporting Items for Systematic Reviews and Meta-Analyses

-5Section/topic	#	Checklist item	Reported on page #
**TITLE**			
Title	1	Identify the report as a systematic review, meta-analysis, or both.	Title
**ABSTRACT**			
Structured summary	2	Provide a structured summary including, as applicable: background; objectives; data sources; study eligibility criteria, participants, and interventions; study appraisal and synthesis methods; results; limitations; conclusions and implications of key findings; systematic review registration number.	Abstract
**INTRODUCTION**			
Rationale	3	Describe the rationale for the review in the context of what is already known.	Introduction
Objectives	4	Provide an explicit statement of questions being addressed with reference to participants, interventions, comparisons, outcomes, and study design (PICOS).	Introduction
**METHODS**			
Protocol and registration	5	Indicate if a review protocol exists, if and where it can be accessed (e.g., Web address), and, if available, provide registration information including registration number.	NA
Eligibility criteria	6	Specify study characteristics (e.g., PICOS, length of follow-up) and report characteristics (e.g., years considered, language, publication status) used as criteria for eligibility, giving rationale.	Methods
Information sources	7	Describe all information sources (e.g., databases with dates of coverage, contact with study authors to identify additional studies) in the search and date last searched.	Methods
Search	8	Present full electronic search strategy for at least one database, including any limits used, such that it could be repeated.	Methods
Study selection	9	State the process for selecting studies (i.e., screening, eligibility, included in systematic review, and, if applicable, included in the meta-analysis).	Methods

Data collection process	10	Describe method of data extraction from reports (e.g., piloted forms, independently, in duplicate) and any processes for obtaining and confirming data from investigators.	Methods
Data items	11	List and define all variables for which data were sought (e.g., PICOS, funding sources) and any assumptions and simplifications made.	Methods
Risk of bias in individual studies	12	Describe methods used for assessing risk of bias of individual studies (including specification of whether this was done at the study or outcome level), and how this information is to be used in any data synthesis.	Methods
Summary measures	13	State the principal summary measures (e.g., risk ratio, difference in means).	Methods
Synthesis of results	14	Describe the methods of handling data and combining results of studies, if done, including measures of consistency (e.g., I^2) for each meta-analysis.	Methods

Section/topic	#	Checklist item	Reported on page #
Risk of bias across studies	15	Specify any assessment of risk of bias that may affect the cumulative evidence (e.g., publication bias, selective reporting within studies).	Methods
Additional analyses	16	Describe methods of additional analyses (e.g., sensitivity or subgroup analyses, meta-regression), if done, indicating which were pre-specified.	Methods
**RESULTS**			
Study selection	17	Give numbers of studies screened, assessed for eligibility, and included in the review, with reasons for exclusions at each stage, ideally with a flow diagram.	Results
Study characteristics	18	For each study, present characteristics for which data were extracted (e.g., study size, PICOS, follow-up period) and provide the citations.	Results
Risk of bias within studies	19	Present data on risk of bias of each study and, if available, any outcome level assessment (see item 12).	Results
Results of individual studies	20	For all outcomes considered (benefits or harms), present, for each study: (a) simple summary data for each intervention group (b) effect estimates and confidence intervals, ideally with a forest plot.	Results

Synthesis of results	21	Present results of each meta-analysis done, including confidence intervals and measures of consistency.	Results
Risk of bias across studies	22	Present results of any assessment of risk of bias across studies (see Item 15).	Results
Additional analysis	23	Give results of additional analyses, if done (e.g., sensitivity or subgroup analyses, meta-regression [see Item 16]).	Results
**DISCUSSION**			
Summary of evidence	24	Summarize the main findings including the strength of evidence for each main outcome; consider their relevance to key groups (e.g., healthcare providers, users, and policy makers).	Discussion
Limitations	25	Discuss limitations at study and outcome level (e.g., risk of bias), and at review-level (e.g., incomplete retrieval of identified research, reporting bias).	Discussion
Conclusions	26	Provide a general interpretation of the results in the context of other evidence, and implications for future research.	Discussion
**FUNDING**			
Funding	27	Describe sources of funding for the systematic review and other support (e.g., supply of data); role of funders for the systematic review.	Acknowledgement

From: Moher D, Liberati A, Tetzlaff J, Altman DG, The PRISMA Group (2009). Preferred Reporting Items for Systematic Reviews and Meta-Analyses: The PRISMA Statement.PLoS Med 6(6): e1000097. doi:10.1371/journal.pmed1000097

For more information, visit:**www.prisma-statement.org**.
